# Neurographic Evidence of Inflammatory Polyneuropathies in Peri-COVID-19 Circumstances and Their Relationship With Acute Disease Severity and Inflammatory Storm

**DOI:** 10.7759/cureus.23517

**Published:** 2022-03-26

**Authors:** Nareen H Hasrat, Haithem J Kadhum, Ali R Hashim, Zaineb A Yakob, Lana A Kadhim, Hassan A Farid

**Affiliations:** 1 Neurophysiology, College of Medicine, University of Basrah, Basra, IRQ; 2 Medical Physiology, College of Medicine, University of Basrah, Basra, IRQ; 3 Neurology, College of Medicine, University of Basrah, Basra, IRQ; 4 Neurophysiology, Basrah Health Directorate, Al-Sadr Teaching Hospital, Basrah, IRQ

**Keywords:** chronic inflammatory demyelinating polyneuroradiculopathy (cidp), guillain-barre syndrome (gbs), electromyography (emg), nerve conduction studies (ncs), neuropathy, covid-19, neurophysiology

## Abstract

Recently, there has been increasing evidence among people infected with coronavirus disease 2019 (COVID-19) of being diagnosed with the typical acute post-infectious inflammatory polyneuroradiculopathy that was formerly known as Guillain-Barré syndrome (GBS), and it is not uncommon that some of them develop chronic inflammatory demyelinating polyneuroradiculopathy (CIDP). However, there is still a large debate and controversy about the link between COVID-19 and polyneuropathy. As a result, a multicentric retrospective cohort study was conducted in Basrah Governorate in the south of Iraq that included 2240 patients over a period of six months. Of those, 1344 patients had a history of COVID-19 in the previous year, and 1.14% of them developed inflammatory polyneuropathy, while only 0.29% (896 patients) of those with no history of COVID-19 had developed inflammatory polyneuropathy. This difference is highly significant, with a relative risk equal to six. The majority of the inflammatory polyneuropathy (44.4%) was diagnosed four to 12 weeks after the COVID-19 infection, with GBS being the most common type (72.2% of cases). Moreover, the nerve conduction velocity, the distal latency, and the amplitude of the most studied nerves were slower, more prolonged, and lower, respectively, among the COVID-19 groups compared with the non-COVID-19 group. Furthermore, there is an inverse correlation between the nerve conduction velocity in the majority of studied nerves and certain inflammatory biomarkers, such as serum ferritin, interleukin-6, and c-reactive protein. Although the occurrence of inflammatory polyneuropathy is more common among the less severe groups of COVID-19, if it occurs in the severe groups, it shows a more aggressive presentation.

## Introduction

Neuropathy is a condition in which one or more nerves are damaged or dysfunctional, resulting in tingling, or numbness, muscle weakness, pain in the affected region, and autonomic symptoms such as sphincter malfunction or orthostatic hypotension [1]. Furthermore, neuropathy can affect a single nerve (mononeuropathy) or a group of nerves in a confined patch asymmetrically, which is known as multifocal neuropathy (mononeuritis multiplex), or several peripheral nerves throughout the body, symmetrically and bilaterally (polyneuropathy). Moreover, it might be acute (less than four weeks), sub-acute (between four and eight weeks), or chronic (more than eight weeks) [2]. On the other hand, neuropathies can be classified in a variety of ways, one of which is based on the sites that are primarily affected in nerve cells: thereby, the axon, which is known as axonopathies, such as in diabetes mellitus related neuropathy or uremia; and foremost, the myelin sheath, which was actually identified as myelinopathy, such as in Guillain-Barré syndrome (GBS) or chronic inflammatory demyelinating polyneuropathy, which is known as chronic inflammatory demyelinating polyneuroradiculopathy (CIDP) [3].

A new disease known as novel coronavirus disease or COVID-19, driven by the virus severe acute respiratory syndrome coronavirus 2 (SARS-CoV-2), emerged in Wuhan, China, in December 2019, with pneumonia-like features [4]. Although most studies focused on respiratory problems during the pandemic, many people also had issues with other systems, including the nervous system [5]. SARS-CoV-2 infections produce neurological symptoms in about 40% of patients involving the central and peripheral nervous systems [6]. According to Basrah-based research, COVID-19 neurological sequelae were found in 60.7% of patients, 10-20% suffering from limb paralysis and peripheral sensory loss [7]. Furthermore, it has become clear that COVID-19-related disabling symptoms in some patients might persist for weeks or even months, and those symptoms in some of these people never go away. Surprisingly, 60 days following the onset of the first symptom, only 13% of previously inpatient COVID-19 individuals were utterly free of any COVID-19-related symptoms. In contrast, 32% reported one or two symptoms, and 55% had three or more symptoms. These symptoms include both non-neurological features, such as fatigue, joint pain, dyspnea, chest pain, cough, sore throat, red eyes, diarrhea, and loss of appetite, as well as neurological features, such as headache, dysgeusia, vertigo, myalgia, and anosmia. Post-COVID-19 syndrome is the name given to this disease or event [8]. In addition, according to a study conducted in Basrah, 62.3% of patients acquire post-COVID-19 syndrome after recovering from an acute infection and complaining from generalized fatiguability, joint and muscle ache, hyposmia, cognitive problems, and even serious neurological disorders, such as stroke as well as other non-neurological sequelae, such as new diabetes mellitus, hypothyroidism, myocardial infarction, and hair loss [9]. Moreover, peripheral neuropathies, particularly inflammatory neuropathies in the post-COVID-19 era, were reported among COVID-19 individuals, according to evidence from numerous studies [10-12].

Electromyography (EMG) and nerve conduction studies (NCS) are peripheral nervous system diagnostic techniques routinely employed in hospitals with clinical neurophysiology services. They are beneficial in evaluating diseases of the muscles, nerves, and nerve roots. In such examinations, the electrical activity of muscles and its transmission across nerves in the limbs are studied [13]. It is critical to emphasize the topic of COVID-19-related inflammatory neuropathy and demonstrate an easy and feasible method for diagnosis and follow-up using electrodiagnostic methods, such as the EMG and NCS to provide clinical recognition, proper care, and rapid therapeutic interventions to avoid further health problems, particularly those that can be life-threatening due to the possibility of respiratory failure, which is primarily associated with GBS. The goal of this study was to show the burden of inflammatory polyneuropathy in patients who have had a preceding COVID-19 infection and examine the NCS and EMG abnormalities in these instances and their association with the respiratory illness severity and inflammatory cytokine release syndrome.

## Materials and methods

A multicentric retrospective cohort study was conducted in Basrah Governorate in the south of Iraq on 2240 patients who attended the neurology unit in Basrah University Teaching Hospital and neurophysiology outpatient clinic in both Al-Sadr Teaching Hospital and Basrah Specialized Children's Hospital; it included the whole number of patients attending these three units for six months from July 1, 2021, to January 1, 2022. This study is a preliminary part of a future case-control study that will be conducted in 2022 to assess the neurophysiological changes in acute and long-term COVID-19 circumstances, whether neurologically symptomatic or not [14]. The participants were subdivided into two groups. The first group (1344 patients) includes those who have had a history of confirmed COVID-19 in the previous year and in whom the diagnosis of COVID-19 was made by the criteria of the European Center of Disease Control (ECDC) which diagnosed COVID-19 by positive polymerase chain reaction (PCR) and/or chest computed tomography (CT) showing peripheral bilateral ground-glass opacities in the presence of strong clinical, serological or epidemiological suspicions [15]. The second group of 896 patients includes those with a negative history of COVID-19. The processing of data recruitment was started by active surveying and identifying the positive cases of GBS and CIDP, which were based on the patients' medical records. Then, those patients were contacted through their telephone numbers to complete the required parts of information concerning their detailed history of COVD-19 infection, the clinical presentation of the recent neurological illness, and the neurophysiological findings by EMG and NCS, as well as the examination findings from the records, in addition to giving their consent for study participation.

The duration after recovery from COVID-19 infection, which is known as "long COVID-19," is defined according to the National Institute of Health and Care Excellence (NICE) guidelines and is further subdivided into "ongoing COVID-19" from four weeks to 12 weeks and "post-COVID-19," in which the symptoms last after 12 weeks. While the "acute COVID-19" includes those during the first four weeks of illness [16]. Acute COVID-19 illness is classified as mild to moderate according to the National Institute of Health's severity classification, which involves asymptomatic patients and those with only respiratory symptoms or a positive chest computed tomography revealing less than 50% lung involvement, a normal respiratory rate, and blood oxygenation of more than 94%. Patients with respiratory distress, as evaluated by tachypnea or oxygen saturation of less than 94%, or a chest computed tomography with much more than 50% lung involvement, as well as those with a history of respiratory failure, cytokine release syndrome, or multiorgan failure, fall into the severe to critical category [17]. The presence of a proinflammatory or cytokine storm (also called cytokine release syndrome), which is the body's hyper-inflammatory immune response evidenced by extreme respiratory distress, was suggested by laboratory findings such as increased serum ferritin as well as interleukin-6 titers and the need for non-invasive techniques such as continuous positive airway pressure (CPAP) or invasive procedures such as mechanical ventilation via endotracheal intubation [18].

The diagnosis of GBS and CIDP is clinically suggestive and neurophysiologically validated by nerve conduction studies (NCS) and electromyography (EMG) based on the criteria that are shown in Table 1 (modified Albers and Kelly criteria) and the normal values of the nerve conduction study parameters are listed in Table 2 [19].

**Table 1:** The diagnostic criteria of neuropathy. GBS: Guillain-Barré syndrome; CIDP: chronic inflammatory demyelinating polyneuropathy

Disorder	Criteria for diagnosis
Polyneuropathy	More than two nerves, symmetrically and bilaterally.
Myelinopathy	Marked decreased nerve conduction velocity below 75% of the lower limit of normal with marked prolongation in the distal latency more than 130% of the upper limit of normal, and normal or mild decline in the amplitude.
Axonopathy	Decrease in the amplitude with normal or mild decrease in the nerve conduction velocity but never below 75% of the lower limit of normal and normal or mild prolongation in the distal latency but never above 130% of the upper limit of normal.
GBS	Three of the following criteria in motor nerves: prolong distal latencies > 115% if normal amplitude or > 125% if decrease amplitude below the lower limit of normal in two or more nerves not at entrapment sites. Conduction velocity slowing < 90% if amplitude > 50% of the lower limit of normal and < 80% of the lower limit of normal if amplitude < 50% of the lower limit of normal in two or more nerves do not cross entrapment sites. Prolong late responses (F wave and H reflex) > 125% of the upper limit of normal in one or more nerves. Conduction block (which is either unequivocal if the proximal to distal amplitude ratio < 0.5 or possible if the ratio < 0.7) and temporal dispersion (proximal to distal duration > 1.15) in one or more nerves.
CIDP	Three of the following criteria in motor nerves: prolong distal latencies > 130% of the upper limit of normal in two or more nerves not at entrapment sites. Conduction velocity slows to < 75% of the lower limit of normal in two or more nerves that do not cross the entrapment site. Prolonged late responses (F wave and H reflex) > 130% of the upper limit of normal in one or more nerves. Conduction block (which is either unequivocal if the proximal to distal amplitude ratio < 0.5 or possible if the ratio < 0.7) and temporal dispersion (proximal to distal duration > 1.15) in one or more nerves.

**Table 2:** The normal reference ranges of the parameters in the nerve conduction study *The recording muscles are abductor pollicis brevis for median nerve, abductor digiti minimi for ulnar nerve, extensor digitorum brevis for peroneal nerve, and abductor hallucis brevis for tibial nerve. **The primary demyelination cut-off level is considered below 35 m/s for the upper limbs and 30 m/s for the lower limbs.

Nerve	Distal latency (ms)		Amplitude (mv)		Conduction velocity (m/s)	
	Right	Left	Right	Left	Right	Left
The motor nerves*^,^ **						
Median	≤ 4.4	≤ 4.4	≥ 4	≥ 4	≥ 49	≥ 49
Ulnar	≤ 3.3	≤ 3.3	≥ 6	≥ 6	≥ 49	≥ 49
Peroneal	≤ 6.6	≤ 6.6	≥ 2	≥ 2	≥ 44	≥ 44
Tibial	≤ 5.8	≤ 5.8	≥ 4.0	≥ 4.0	≥ 41	≥ 41
The sensory nerves**						
Ulnar	≤ 3.1	≤ 3.1	≥ 17	≥ 17	≥ 50	≥ 50
Sural	≤ 4.4	≤ 4.4	≥ 6.0	≥ 6.0	≥ 40	≥ 40
F wave latency (ms)			-	-	-	-
Ulnar	≤ 32	≤ 32	-	-	-	-
Tibial	≤ 56	≤ 56	-	-	-	-

The study results were analyzed using the computerized Statistical Package for Social Science (SPSS) version 26 (Armonk, NY: IBM Corp.) software. The numerical data were tabulated as mean and standard deviation (SD), and the two-sample Student t-test was used to compare the two groups. The qualitative data was tallied as a percentage and analyzed using the chi-square or Fisher's exact test. A p-value of equal or less than 0.05 is regarded as statistically significant, and a value of equal or less than 0.01 is considered highly significant. Furthermore, the correlation between the nerve condition study parameters and various blood biomarkers during the COVID-19 illness was analyzed using the Pearson correlation test.

## Results

The study involved 2240 patients. Of those, 1344 had a history of COVID-19 infection and 18 cases from this group (1.14%) developed inflammatory polyneuropathy (GBS and CIDP). Only two patients (0.29%) of those without a history of COVID-19 (896 patients) developed inflammatory polyneuropathy, and this difference is statistically significant (p < 0.01) (Table 3).

**Table 3:** The relationship between inflammatory neuropathy and COVID-19 infection GBS: Guillain-Barré syndrome; CIDP: chronic inflammatory demyelinating polyneuropathy; COVID-19: coronavirus disease 2019

Inflammatory neuropathy	History of COVID-19	No history of COVID-19	Total	p-Value
Developed GBS/CIDP	18 (1.14 %)	2 (0.29 %)	20	0.005
Not developed GBS/CIDP	1326 (98.86 %)	894 (99.71 %)	2220	
Total	1344	896	2240	

Regarding the demographic characteristics of the patients enrolled in the study, for the COVID-19-related neuropathy group, the mean age was 53.34 years, in comparison with 33.5 years as the mean age for those who did not have a history of COVID-19, and this difference is statistically significant (p < 0.05). As for gender distribution, males were predominant (55.6%) among the COVID-19 polyneuropathy, in contrast to the non-COVID-19 group, which had only two female cases (100%). Furthermore, the majority of the COVID-19 polyneuropathy cases were from rural origins (61.1%) and most of them had a history of chronic illnesses (55.6%), which was in the form of recent (at time of COVID-19 illness) diabetes mellitus in three cases, hypertension in seven cases, and one case with a history of ischemic cardiac disease, but these parameters made no statistical difference when compared with the polyneuropathy cases from the non-COVID-19 groups (Table 4). 

**Table 4:** The characteristics of the patients with inflammatory polyneuropathy (n=20) *Difference between the first and second age groups. **Difference between the first and third age groups. ***Difference between the second and third age groups. SD: standard deviation; DM: diabetes mellitus; HTN: hypertension; IHD: ischemic heart disease; COVID-19: coronavirus disease 2019

Demographic characteristics		COVID-19 group (n=18)	Non-COVID-19 group (n=2)	p-Value
Age/years	Mean age	53.34 (SD 15.76)	33.5 (SD 4.94)	0.03
	< 35	3 (16.7%)	1 (50%)	0.50^*^, 0.40^**^, 1.00^***^
	35-65	9 (50%)	1 (50%)	
	> 65	6 (33.3%)	Zero	
Sex	Male	10 (55.6%)	Zero	0.47
	Female	8 (44.4%)	2 (100%)	
Residency	Urban	7 (38.9%)	2 (100%)	0.18
	Rural	11 (61.1%)	Zero	
Chronic illnesses	Present	10 (55.6%)	Zero	0.47
	Absent	8 (44.4%)	2 (100%)	
	DM	3 (16.67%)	Zero	1.00
	HTN	7 (38.89%)	Zero	0.52
	IHD	1 (5.56%)	Zero	1.00

Based on the results of the nerve conduction study with clinical correlation regarding the time of presentation, neurological manifestation, and clinical progression, GBS was diagnosed in 13 (72.2%) of COVID-19-related neuropathy and in one case (50%) of non-COVID-19-related polyneuropathy, while the remaining cases in both groups were diagnosed as CIDP. Regarding the nerve involvement, 14 (77.8%) of cases among the COVID-19 group showed mixed motor and sensory nerve damage, and only four (22.2%) of these cases had pure motor nerve dysfunction, but for the non-COVID-19-related neuropathy, both two cases (100%) showed mixed involvement. From the pathological point of view, the majority of COVID-19 inflammatory polyneuropathy were of demyelination pattern, with only one case (5.6%) of an axonal variant of GBS, and both the two cases of polyneuropathy in the non-COVID-19 group demonstrated myelinopathy. Still, none of the previously mentioned results showed a statistical difference between the two study groups (Table 5). 

**Table 5:** The diagnosis of inflammatory neuropathy according to the nerve conduction studies GBS: Guillain-Barré syndrome; CIDP: chronic inflammatory demyelinating polyneuropathy; COVID-19: coronavirus disease 2019

Classification of neuropathy		COVID-19 group	Non-COVID-19 group	p-Value
Diagnosis	GBS	13 (72.2%)	1 (50%)	0.52
	CIDP	5 (27.8%)	1 (50%)	
Function	Mixed motor and sensory	14 (77.8%)	2 (100%)	1.00
	Pure Motor	4 (22.2%)	Zero	
Pathology	Demyelination	17 (94.4%)	2 (100%)	1.00
	Axonopathy	1 (5.6%)	Zero	
Total		18	2	20

Among the COVID-19-related neuropathy patients, the neurological manifestation started to develop after approximately 16 days of illness, but the diagnosis of inflammatory polyneuropathy based on NCS and EMG findings was elicited in less than four weeks for three cases, within three months for eight cases, and after four months for seven cases. In almost all cases, except one, there was limb weakness, whether it was in the lower limbs (61.1%) only or both upper and lower (33.3%), and the majority (72.2%) showed both proximal and distal patterns of weakness, and in all cases of weakness (94.3%), it had an ascending and symmetrical pattern. In the majority (61.1%) of the cases, paresthesia and numbness affected the lower limbs only, and lower back pain was present in 83.31% of them. Furthermore, neuropathic burning pain was manifested in 16 cases, and it mainly affected the lower limbs (55.6%), while myalgia was discovered in 14 cases, and it affected both the upper and lower limbs in 44.4% of cases. Moreover, half of the patients had ataxia and unsteadiness, while 83.3% of them did not develop any sphincter dysfunction. For bulbar involvement, dysphagia and dysarthria were equally present in only four (22.2%) cases, and only three (16.6%) were reported to have dyspnea. Facial palsy was not reported in this cohort. Regarding the details of the motor system neurological assessment, hypotonia was present in 17 cases, mainly in the lower limbs. The decrement in power grades was most prevalent in the lower limbs. Additionally, hyporeflexia or even absent reflexes were found in the knee and ankle jerks of 17 cases (99.4%), while in about half of the cases in the upper limb reflexes. Furthermore, lumbosacral spinal MRI was done for 11 patients out of 18. In nine patients, it was normal, and in the other two, it showed mild disc herniation in the lower lumbar discs. Unfortunately, cerebrospinal fluid (CSF) analysis was done only for two patients. One showed cytoalbuminologic disassociation and the other was normal, but the remaining patients refused to have this procedure. Moreover, all the 18 COVID-19 patients received intravenous immunoglobulin (IVIG) once the diagnosis of GBS or CIDP was confirmed, and only one of the cases of CIDP failed to show any improvement and developed continuous deterioration and progression even with recurrent IVIG therapy. Therefore, she received plasmapheresis and was later kept on azathioprine, but she died soon after that from pneumonia-related sepsis and respiratory failure. Regarding the outcome, complete recovery is documented in only two cases, while the other cases reported partial or incomplete resolution of weakness. All these results are expressed in Table 6.

**Table 6:** The clinical features, diagnostic test, and treatment options of COVID-19-related inflammatory neuropathy cases SD: standard deviation; CSF: cerebrospinal fluid; MRI: magnetic resonance image; IVIG: intravenous immunoglobulin; COVID-19: coronavirus disease 2019

Clinical features				Mean (SD)/frequency (%)
Time of onset of neurological complaint in relation to COVID-19 infection				16.34 ± 6.69
Time of diagnosis (total duration)	Acute (<4 weeks)			3 (16.7%)
	Ongoing (4-12 weeks)			8 (44.4%)
	Post-COVID-19 (>12 weeks)			7 (38.9%)
Limb weakness	No weakness			1 (5.6%)
	Upper only			0
	Lower only			11 (61.1%)
	Both upper and lower limbs			6 (33.3%)
	Proximal only			3 (16.7%)
	Distal only			1 (5.6%)
	Both proximal and distal			13 (72.2)
	Ascending			17 (94.4%)
	Descending			Zero
	Symmetrical			17 (94.4%)
	Asymmetrical			0
Paresthesia and numbness	Upper limb			0
	Lower limb			11 (61.1%)
	Both upper and lower limbs			6 (33.3%)
Low back pain	Absent			3 (16.7%)
	Present			15 (83.3%)
Neuropathic burning pain	Absent			2 (11.1%)
	Upper limb			0
	Lower limb			10 (55.6%)
	Both upper and lower limbs			6 (33.3%)
Myalgia	Absent			4 (22.2%)
	Upper limb			0
	Lower limb			6 (33.3%)
	Both upper and lower limbs			8 (44.4%)
Ataxia	Absent			9 (50%)
	Present			9 (50%)
Sphincter dysfunction	Absent			15 (83.3%)
	Bladder dysfunction			3 (16.7%)
	Bowel dysfunction			1 (5.6%)
Dysphagia/dysarthria	Absent			14 (77.8%)
	Present			4 (22.2%)
Dyspnea (respiratory muscle involvement)	Absent			15 (83.4%)
	Present			3 (16.6%)
Facial palsy				0
Tone	Upper limbs	Normal		8 (44.4%)
		Hypotonia		10 (55.6%)
		Hypertonia		0
	Lower limbs	Normal		1 (5.6%)
		Hypotonia		17 (94.4%)
		Hypertonia		0
Power	Upper limbs	Grade 5		6 (33.3%)
		Grade 4		8 (44.4%)
		Grade 3		2 (11.1%)
		Below Grade 3		2 (11.1%)
	Lower limbs	Grade 5		1 (5.6%)
		Grade 4		5 (27.8%)
		Grade 3		7 (38.8%)
		Below Grade 3		5 (27.8%)
Reflexes	Upper limbs	Normal		8 (44.4%)
		Hypo/areflexia		10 (55.6%)
	Lower limbs	Normal		1 (5.6%)
		Hypo/areflexia		17 (94.4%)
CSF analysis	Done	Normal		1 (5.6%)
		Cytoalbuminologic disassociation		1 (5.6%)
	Not done			16 (88.8%)
Lumbosacral spinal MRI	Done		Normal	9 (50.0%)
			Mild discs prolapse	2 (11.1%)
	Not done			7 (38.8%)
Treatment options	IVIG			18 (100.0%)
	Plasmapheresis			1 (5.5%)
Outcome	Complete recovery			2 (11.1%)
	Partial response			15 (83.3%)
	Death			1 (5.5%)

According to the findings in the NCS, both left and right median motor nerves had prolonged distal latency (DL), lower amplitude, slower nerve conduction velocity (NCV), and a higher rate of conduction block (CB) in COVID-19-related polyneuropathy in comparison with non-COVID-19-related neuropathy (Table 7). An absent response was also noticed in one of the cases in the COVID-19 group. For left and right ulnar motor nerves, the DL was also prolonged in the COVID-19 group, and the NCV was lower, but the amplitude was higher. Again, CB was more frequent among COVID-19 cases and the F wave latency was more prolonged in this group when compared with the non-COVID-19 cases. Regarding the motor nerves in the lower limbs, the parameters of the left and right peroneal nerves were also more affected in the COVID-19 group except for amplitude, and the response was absent in seven cases in this group. Regarding the tibial nerve parameters, the left tibial nerve seems to be more affected in the non-COVID-19 groups. Despite four cases in the other group failing to demonstrate response, the right tibial appeared to be more affected in the COVID-19 group in regards to DL and amplitude, but the conduction velocity was faster. Again, four cases were documented with no response. Furthermore, F wave latency in both the right and left tibial nerves was more prolonged in the COVID-19 group. On the other hand, for sensory nerve conduction studies, the DL, amplitude, and NCS in the non-COVID-19 group appeared to be more affected in comparison with COVID-19-related neuropathy cases. Finally, sural nerve parameters showed no response in the two non-COVID-19 inflammatory polyneuropathies and half the cases in the COVID-19-related polyneuropathy. None of the above-mentioned results showed significant statical differences between the two groups.

**Table 7:** The parameters of motor and sensory nerves in COVID-19 and non-COVID-19 groups DL: distal latency; amp: amplitude; NCV: nerve conduction velocity; COVID-19: coronavirus disease 2019

Nerve/parameter			Non-COVID-19 group (n=2)	COVID-19-group (n=18)	p-Value
Median motor	Left	DL	5.55 ± 2.47	8.36 ± 3.97	0.34
		Amp	3.30 ± 0.57	2.92 ± 1.17	0.65
		NCV	41.10 ±14.23	38.01 ± 7.95	0.63
		Conduction block	0	6 (35.37%)	0.47
		Absent response	0	1 (5.60%)	0.90
	Right	DL	5.25 ± 1.25	8.25 ± 4.45	0.36
		Amp	3.80 ± 0.99	3.14 ± 1.65	0.59
		NCV	39.35 ± 8.41	39.21 ± 7.61	0.98
		Conduction block	1 (50.00%)	2 (11.10%)	0.28
		Absent response	0	1 (5.60%)	0.90
Ulnar motor	Left	DL	4.60 ± 2.55	5.35 ± 1.80	0.59
		Amp	2.40 ± 1.41	3.58 ± 1.82	0.39
		NCV	45.80 ± 13.44	36.85 ± 9.64	0.24
		Conduction block	1 (50.00%)	7 (38.90%)	0.65
		Absent response	0	1 (5.60%)	0.90
		F wave	37.75± 5.87	43.6 ± 7.10	0.28
	Right	DL	4.20 ± 1.84	5.58 ± 1.85	0.33
		Amp	2.75 ± 0.92	3.45 ± 1.85	0.61
		NCV	45.15 ± 14.21	37.45 ± 8.91	0.28
		Conduction block	0	5 (27.80%)	0.55
		Absent response	0	1 (5.60%)	0.90
		F wave	38.90 ± 6.51	44.36 ± 7.67	0.35
Peroneal motor	Left	DL	8.90	9.22 ± 3.94	0.94
		Amp	1.10	1.61 ± 0.97	0.62
		NCV	29.10	27.87 ± 9.20	0.90
		Conduction block	0	1 (5.60%)	0.90
		Absent response	1 (50.00%)	7 (38.90%)	0.65
	Right	DL	4.70	10.23 ± 3.58	0.17
		Amp	1.40	1.6 ± 1.07	0.86
		NCV	46.10	27.56 ± 10.03	0.10
		Conduction block	0	2 (11.10%)	0.80
		Absent response	1 (50.00%)	7 (38.90%)	0.65
Tibial motor	Left	DL	10.50	8.93 ± 3.39	0.66
		Amp	2.90	2.84 ± 1.69	0.97
		NCV	26.50	30.38 ± 6.52	0.57
		Conduction block	0	1 (5.60%)	0.90
		Absent response	1 (50.00%)	4 (22.23%)	0.44
		F wave	71.3 ± 23.62	81.22 ± 10.97	0.29
	Right	DL	9.90	8.98 ± 2.13	0.68
		Amp	3.50	2.54 ± 1.66	0.58
		NCV	28.10	29.35 ± 7.75	0.87
		Conduction block	0	1 (5.60%)	0.90
		Absent response	1 (50.00%)	4 (22.20%)	0.44
		F wave	67 ± 16.26	79.63 ± 11.35	0.17
Ulnar sensory	Left	DL	4.90	3.7 ± 1.09	0.33
		Amp	3.50	11.46 ± 6.40	0.26
		NCV	26.10	35.72 ± 9.89	0.37
		Absent response	1 (50.00%)	7 (38.90%)	0.65
	Right	DL	4.50	3.8 ± 1.13	0.56
		Amp	4.40	11.35 ± 7.34	0.38
		NCV	29.30	34.88 ± 10.01	0.60
		Absent response	1 (50.00%)	6 (33.30%)	0.58
Sural sensory	Left	DL	-	3.24 ± 0.73	-
		Amp	-	8.54 ± 2.62	-
		NCV	-	44.78 ± 9.24	-
		Absent response	2 (100%)	9 (50.00%)	0.28
	Right	DL	-	3.11 ± 0.61	-
		Amp	-	8.93 ± 3.64	-
		NCV	-	45.87 ± 7.07	-
		Absent response	2 (100%)	9 (50.00%)	0.28

The characteristics of COVID-19 infection for those with inflammatory polyneuropathy are summarized in Table 8 and showed that most of the neuropathy cases (72.2%) had a history of mild-to-moderate infection and about 83% had no history of the hyperacute inflammatory cytokine storm. Moreover, most of the cases (61.1%) were treated at home and did not require hospital admission. Regarding the duration of respiratory illness, in approximately 55% of the cases, the illness did not extend beyond two weeks. Furthermore, the oxygen saturation was normal in more than 70% of the cases. The blood biomarkers of these cases showed mild-to-moderate elevation in serum ferritin, C-reactive protein, lactate dehydrogenase (LDH), and interleukin 6 (IL-6) but a normal mean for the N/L ratio. Regarding the diagnostic confirmation of acute COVID-19 infection among GBS and CIDP cases, positive PCR for SARS-CoV-2 was demonstrated in all 18 cases, and about 40% of the cases had evidence of lung involvement by chest CT scan. Furthermore, none of the patients who developed GBS or CIDP had received the COVID-19 vaccination or any recent vaccination.

**Table 8:** The COVID-19 features among COVID-19-related inflammatory neuropathy cases PCR: polymerase chain reaction; GBS: Guillain-Barré syndrome; CIDP: chronic inflammatory demyelinating polyneuropathy; COVID-19: coronavirus disease 2019

COVID-19 features		Mean (SD)/frequency (%)
The severity of COVID-19	Mild-moderate	13 (72.2%)
	Sever-critical	5 (27.8%)
The occurrence of cytokine storm	Absent	15 (83.3%)
	Present	3 (16.7%)
The history of hospitalization	Home management	11 (61.1%)
	Ward admission	6 (33.3%)
	ICU admission	1 (5.6%)
Duration of respiratory illness	Mean (SD)	10 (4.43)
	<1 week	7 (38.9%)
	1-2 weeks	10 (55.6%)
	>2 weeks	1 (5.6%)
Oxygen saturation during COVID-19 illness	94-100 %	13 (72.2%)
	80-93 %	4 (22.2%)
	70-79 %	1 (5.6%)
Serum ferritin (27-375 ng/mL in men and 12-135 ng/mL in women)		493.11 ± 434.08
C-reactive protein (CRP) (0-5 mg/L)		37.39 ± 24.48
Lactate dehydrogenase (LDH) (135-225 U/L)		400.97 ± 255.48
Interleukin-6 (IL-6) (0-7 pg/mL)		19.23 ± 22.48
Neutrophile-to-lymphocyte ratio (NLR) (0.78-3.53)		3.23 ± 1.68
Positive PCR		18 (100%)
Positive chest CT for typical COVID-19		8 (44.45%)
History of COVID-19 vaccination prior to GBS/CIDP		0

The differences in the motor nerve conduction study parameters based on the disease severity and the occurrence of cytokine storm revealed that for the left median motor nerve, the DL and amplitude are affected more in the mild severity group, but the nerve conduction velocity is slower in the severe group, while for the right median nerve, the DL was also prolonged more in the mild group, but the amplitude and NCV were better than the readings in the severe group (Table 9). In terms of the cytokine storm, the nerve conduction velocities for both the right and left median nerves appear to be more affected in those who have cytokine storms. The ulnar motor nerve study revealed prolonged DL and lower NCV on both sides for the severe group and also for those with cytokine storm, but lower amplitudes were noticed bilaterally in both the mild group and those with no cytokine storm. For upper limb nerves, these differences have not shown statistical significance. Regarding the lower limb motor nerve studies, for the peroneal nerve, more prolonged DL and lower amplitudes were noticed bilaterally among the severe group compared with the mild group, but the NCV was variable as it was lower for the left in the severe group but higher for the right nerve in the same group. Unfortunately, the comparison could not be made regarding the occurrence of cytokine storm as there is only one nerve that shows response among those with cytokine storm and the other two in this group elicit no response, so statistical analysis could not be done. The tibial nerve showed prolonged DL, lower amplitude, and slower NCV on the left side among the severe group, but the comparison among the cytokine storm group was also not applicable due to the absence of response in certain cases. For the right tibial nerve, the only lower mean of the amplitude is noticed in the severe groups and those with the storm, but there is better DL and conduction velocity in the milder group. However, all these results have no statistically significant differences except for right tibial nerve amplitude, which is significantly lower among the cytokine storm group (p=0.04).

**Table 9:** The relationship between nerve conduction study parameters and disease severity DL: distal latency; amp: amplitude; NCV: nerve conduction velocity

Nerve parameter vs. severity			Mild-moderate (n=13)		Severe-critical (n=5)	p-Value	No cytokine storm (n=15)	Cytokine storm (n=3)	p-Value
Median motor	Left	DL		8.42 ± 4.01	8.18 ± 4.46	0.92	8.17 ± 0.98	6.92 ± 4.90	0.60
		Amp		2.82 ± 1.31	3.23 ± 0.41	0.35	2.83 ± 1.22	3.55 ± 0.07	0.43
		NCV		38.67 ± 6.58	35.88 ± 12.49	0.56	38.35 ± 6.26	35.45 ± 21.14	0.88
	Right	DL		8.45 ± 4.59	7.65 ± 4.51	0.77	8.06 ± 4.38	9.75 ± 6.58	0.63
		Amp		3.42 ± 1.68	2.25 ± 1.35	0.23	3.35 ± 1.59	1.55 ± 1.63	0.15
		NCV		40.68 ± 6.89	34.43 ± 8.92	0.16	40.02 ± 6.61	33.15 ± 15.20	0.64
Ulnar motor	Left	DL		5.24 ± 1.52	5.70 ± 2.79	0.67	5.21 ± 1.42	6.45 ± 4.59	0.38
		Amp		3.35 ± 1.85	4.33 ± 1.77	0.37	3.42 ± 1.73	4.75 ± 2.89	0.35
		NCV		38.2 ± 8.64	32.48 ± 12.77	0.31	37.26 ± 8.41	33.80 ± 21.78	0.86
	Right	DL		5.42 ± 1.20	6.07 ± 3.45	0.55	5.33 ± 1.15	7.45 ± 5.30	0.13
		Amp		3.28 ± 1.96	4.00 ± 1.54	0.52	3.31 ± 1.83	4.50 ± 2.40	0.41
		NCV		38.38 ± 8.56	34.42 ± 10.69	0.46	37.58 ± 8.21	36.45 ± 18.03	0.87
Peroneal motor	Left	DL		9.04 ± 4.37	10.00 ± 1.13	0.77	9.22 ± 4.15	9.20	-
		Amp		1.61 ± 1.04	1.60 ± 0.85	0.99	1.67 ± 1.00	1.00	-
		NCV		28.38 ± 9.96	25.60 ± 6.36	0.72	28.55 ± 9.41	21.10	-
	Right	DL		10.16 ± 4.14	10.40 ± 2.04	0.93	10.44 ± 3.70	8.10	-
		Amp		1.75 ± 1.16	1.17 ± 0.85	0.45	1.60 ± 1.13	1.50	-
		NCV		26.94 ± 11.69	29.23 ± 4.30	0.75	27.81 ± 10.53	25.10	-
Tibial motor	Left	DL		8.65 ± 3.73	9.97 ± 1.70	0.57	8.70 ± 3.41	11.90	-
		Amp		2.94 ± 1.85	2.47 ± 1.14	0.69	2.96 ± 1.69	1.20	-
		NCV		30.77 ± 7.07	28.93 ± 4.74	0.68	30.68 ± 6.68	26.40	-
	Right	DL		8.51 ± 2.23	10.15 ± 1.46	0.20	8.78 ± 2.12	10.14 ± 2.47	0.56
		Amp		2.90 ± 1.64	1.65 ± 1.55	0.22	2.90 ± 1.49	0.35 ± 0.07	0.04
		NCV		29.25 ± 9.16	29.6 ± 2.89	0.94	29.31 ± 8.35	29.60 ± 3.82	0.96
Ulnar sensory	Left	DL		3.73 ± 1.07	3.70 ± 1.69	0.97	3.85 ± 1.08	2.50	-
		Amp		11.43 ± 6.09	11.60 ± 10.61	0.98	10.70 ± 6.19	19.10	-
		NCV		34.87 ± 9.98	39.55 ± 12.09	0.57	34.48 ± 9.49	48.10	-
	Right	DL		3.80 ± 1.79	3.83 ± 1.21	0.97	3.91 ± 1.13	2.70	-
		Amp		11.82 ± 7.11	9.93 ± 9.51	0.72	10.48 ± 7.03	20.90	-
		NCV		34.82 ± 10.85	35.07 ± 8.95	0.97	34.01 ± 10.01	44.50	-
Sural sensory	Left	DL		3.16 ± 0.73	3.90	-	3.16 ± 0.73	3.90	-
		Amp		8.75 ± 2.73	6.90	-	8.75 ± 2.73	6.90	-
		NCV		45.51 ± 9.59	38.90	-	45.51 ± 9.59	38.90	-
	Right	DL		2.99 ± 0.52	4.10	-	2.99 ± 0.52	4.10	-
		Amp		9.16 ± 3.83	7.10	-	9.16 ± 3.83	7.10	-
		NCV		47.1 ± 6.46	36.10	-	47.10 ± 6.46	36.10	-

Regarding the sensory nerve conduction study parameters, no significant differences are observed in the DL and amplitude of the left sensory ulnar nerve, but slower conduction velocity is reported among the mild group (Table 9). Still, statistical tests cannot be performed to compare the difference according to cytokine storm status due to the absence of response. In the right sensory ulnar nerve, the DL and amplitude are slightly more affected in the severe group, but the NCV is slightly slower in the milder severity group. These differences have no statistical significance. The differences in the parameters of the sural nerve study could not be assessed statistically due to the absence of response in the severe and cytokine groups.

The correlation between the nerve conduction velocities in the studied nerves with certain inflammatory biomarkers was assessed using the Pearson correlation test (Table 10). Both right and left median nerve conduction velocities were negatively correlated with all the studied biomarkers (c-reactive protein {CRP}, ferritin, LDH, IL-6, and, N/L ratio), and the strongest correlations were detected with IL-6 and N/L ratio, but none of these correlations showed statistical significance. Regarding the ulnar nerve conduction velocities bilaterally, the negative correlation was only detected with the N/L ratio, but no strong association was found. Peroneal and tibial nerves bilaterally showed a similar pattern of median nerves as the NCV negatively correlated with all the studied biomarkers, but the strongest correlation was with CRP and LDH for peroneal nerves and mainly with CRP for the tibial nerve, but none of these results were statistically significant. Consequently, the correlation for sensory nerve conduction velocities showed a positive correlation with the inflammatory biomarkers for the ulnar nerve, with some similarity to the motor component of this nerve, but for the sural nerve, which is again the same as the median, peroneal, and tibial, it showed a negative correlation with all the studied biomarkers, and the strongest correlations were between the N/L ratio and CRP with the left sural nerve conduction velocity.

**Table 10:** The correlation between nerve conduction velocity and blood biomarkers in patients with COVID-19-related inflammatory polyneuropathy (n=18) NCV: nerve conduction velocity; CRP: c-reactive protein; LDH: lactate dehydrogenase; N/L ratio: neutrophile-to-lymphocyte ratio; IL-6: interleukin-6; COVID-19: coronavirus disease 2019

NCV vs. inflammatory biomarkers			CRP	Ferritin	LDH	N/L ratio	IL-6
Median motor	Left	r	-0.19	-0.22	-0.18	-0.37	-0.33
		P	0.47	0.39	0.49	0.14	0.19
	Right	r	-0.16	-0.24	-0.19	-0.40	-0.22
		P	0.54	0.35	0.46	0.11	0.40
Ulnar motor	Left	r	0.06	0.03	0.08	-0.21	-0.01
		P	0.83	0.91	0.74	0.43	0.97
	Right	r	0.10	0.09	0.13	-0.19	0.06
		P	0.70	0.74	0.62	0.47	0.83
Peroneal motor	Left	r	-0.42	-0.33	-0.35	-0.17	-0.24
		P	0.19	0.33	0.29	0.62	0.48
	Right	r	-0.15	-0.23	-0.39	-0.16	-0.09
		P	0.66	0.49	0.24	0.64	0.78
Tibial motor	Left	r	-0.44	-0.23	-0.19	-0.18	-0.19
		P	0.11	0.42	0.51	0.54	0.49
	Right	r	-0.13	-0.04	-0.15	-0.15	-0.03
		P	0.67	0.89	0.62	0.60	0.91
Ulnar sensory	Left	r	0.30	0.48	0.48	0.17	0.42
		P	0.37	0.14	0.13	0.61	0.19
	Right	r	0.13	0.34	0.36	0.03	0.36
		P	0.69	0.29	0.26	0.92	0.25
Sural sensory	Left	r	-0.35	-0.27	-0.28	-0.51	-0.15
		P	0.36	0.48	0.47	0.16	0.70
	Right	r	-0.59	-0.48	-0.56	-0.61	-0.45
		P	0.09	0.19	0.12	0.08	0.22

## Discussion

The relationship between COVID-19 and inflammatory neuropathy is a challenging topic, and noticeably, there is evidence from different works of literature that highlights the increment in the incidence of GBS and CIDP after infection with COVID-19, although the mechanism behind this new phenomenon is still vague. Finsterer et al., in their work in 2021, assumed that the pathogenesis is primarily caused by immune mechanisms or neurotoxic side effects of drugs used to treat COVID-19 symptoms [10]. According to Whittaker et al. in 2020, the spike protein binds to the ACE-2 receptor and the sialic acid component of cell membrane glycoproteins and gangliosides, which indicates that SARS-CoV-2 is neurotropic and has neuroinvasive potential. The virus also creates antibodies that attack neuronal surface glycoproteins, or gangliosides, such as GD1b, GQ1b, and GT1b. Furthermore, dysregulated cytokine and chemokine production exacerbates peripheral demyelination [20]. However, according to research by Uncini et al., autoimmunity does not entirely explain this event because the majority of patients did not respond well to traditional therapy of intravenous immunoglobulin and plasma exchange [21]. This is also the situation for the cases of GBS/CIDP in this study as the majority showed unsatisfactory responses to this therapy (Table 6).

Regardless of the controversy surrounding the causal relationship between inflammatory neuropathy and COVID-19, the noticeably increasing number of GBS and CIDP cases worldwide in the peri-COVID-19 era is becoming a fact. According to our results, we found that COVID-19 increases the risk of GBS and CIDP by six folds more than the normal non-COVID-19 population, which is a highly significant difference (p=0.005), and these findings are approximately consistent with the results of Gigli et al. in 2021, who conducted a review of the published literature on GBS linked with COVID-19 infection and stated that there would be a 5.41-fold increase in GBS cases after COVID-19 infection (Table 3) [22].

Our cohort study shows that inflammatory polyneuropathy patients in the COVID-19 group have a greater mean age than those in the non-COVID-19 group, and this difference is statistically significant. In addition, it reveals a higher prevalence among males and those with concurrent medical illnesses compared to the non-COVID-19 group, which exhibits a female and previously healthy predominance, but this is not statistically significant (Table 4). This finding is in line with Uncini et al.'s review from 2020, which revealed that the median age of patients was 57.5 years and that the majority of patients were men (64.3%) [21]. Also, our findings are similar to those of Zuberbühler et al., who reported in 2021 that male cases accounted for 64.6% of all cases, with a mean age of 56.4 years [23]. Similarly, according to the most recent published literature by Tawakul et al. in 2022, the average age of the patients was 56.6 years, the most common gender was male (59.05% of cases), and only 18.10% of patients had no previous medical history [24].

Based on the diagnostic criteria of neuropathy that were mentioned in Table 1 previously and the normal references mentioned in Table 2, it was found that among the COVID-19 group, GBS was the most common type of inflammatory polyneuropathy, and all the cases were acute inflammatory demyelinating polyneuropathy (AIDP) except one, which was reported as acute motor-sensory axonal neuropathy (AMSAN), and the majority of patients had mixed motor and sensory involvement (Table 5). These results are also consistent with those of Tawakul et al. in 2022; they found that AIDP was the most common type, and classic sensorimotor GBS was the commonest type and accounted for 56.19% of all cases, while pure motor GBS accounted for 17.14% of patients [24].

The majority of inflammatory polyneuropathy was detected between four and 12 weeks following the COVID-19 illness, with the average time of the start of neurological complaints being 16.34 days after the commencement of the illness. After 12 weeks of COVID-19 infection, 38.9% of patients develop inflammatory neuropathy (Table 6). This pattern is comparable to that reported by Tawakul et al. in 2022; they claimed that the average period between the onset of COVID-19 symptoms and the onset of GBS signs was 15.77 days. However, post-infectious patterns were observed in 58.10% of people [24]. Nevertheless, this differs from the findings of Caress et al. in 2020, who found that many patients had GBS within seven days of being infected with COVID-19, while the remaining majority acquired GBS between seven and 28 days after the onset of COVID-19 illness [11].

Regarding the clinical presentation, about 61% of patients had lower limb weakness as the initial presentation, and the weakness, in most cases; affects both proximal and distal muscles (Table 6). There was an ascending pattern in all cases of weakness. The neuropathic pain was also noticed in 61% of cases, with paresthesia and numbness in addition to the presence of lower back pain in about 83% of patients and myalgia reported in 78% of them, while ataxic gait was noticed in half of the cases. Characteristically, 83.3% of the cases had absent sphincter dysfunction at the time of presentation, which favors the diagnosis of inflammatory polyneuroradiculopathy over other root compression causes. Bulbar features are also noticed inform of dysphagia and dysarthria in about 22% of cases. Unfortunately, 16% of subjects developed dyspnea at the time of illness, which may reflect the affection of respiratory muscles or also the bulbar involvement. Additionally, and as expected in the usual GBS and CIDP scenarios, lower limb hyporeflexia is predominant in almost all cases, and upper limb hyporeflexia was detected in 55% of them. Again, these findings have some similarities with other studies that reported the neurological symptoms in COVID-19 patients with GBS were limb weakness (76.19%), paraesthesia or pain (49.52%), and gait impairment (25.71%). Patients with bulbar symptoms accounted for 18.10% of all patients, and hyporeflexia was found in 88% of cases [24], and Uncini et al. discovered that the most clinically significant symptoms are limb weakness (76.2%), hyporeflexia (80.9%), and sensory disturbances (66.7%) [21].

Concentrating on the parameters of the nerve conduction studies, our study found that the distal latencies were more prolonged among the COVID-19 group than the non-COVID-19 group in most of the studied nerves, including motor median, ulnar, and peroneal nerves, except tibial and sensory ulnar nerves, which show more prolonged latency among non-COVID-19 groups (Table 7). For the action potential amplitudes, they were lower among the COVID-19 group for the motor median and tibial nerves bilaterally. Still, they were higher among the same group for both motor and sensory ulnar nerves and peroneal nerves. Regarding the nerve conduction velocities, they showed close similarities with the distal latencies as they were slower in the motor median, ulnar, and peroneal motor nerves but faster in the tibial nerves and ulnar sensory nerves. As is typical for sural nerves, they were spared in half of the cases in the COVID-19 groups, and this is consistent with the typical GBS diagnosis, although the other 50% reported absent responses [19]. Moreover, the conduction block at non-entrapment sites is reported in 5-40% of the involved nerves in the cases, and it is more commonly found among the motor ulnar and median nerves. Furthermore, the absence response is also noticed in many nerves among the COVID-19 groups with the same range as the conduction block (5-40%) but it is commonly noticed in peroneal motor nerves and ulnar sensory nerves. Actually, these findings are the standard for the diagnosis of demyelinating neuropathy, as it is characterized by prolonged distal latency, slower conduction velocity, and mildly lower amplitude. Although these were more evident among the COVID-19 group than the non-COVID-19 group, this was not a statistically significant difference, as the non-COVID-19 group had a small number of cases. These findings are consistent with the reports of Yaranagula and Koduri in 2021 [25].

Returning to the details of COVID-19 infection among the neuropathy cases, we found that the majority (72.2%) had mild-to-moderate severity at the time of respiratory illness and 83.3% did not report having a cytokine storm. Most of these cases (61.1%) did not require hospital admission at the time of acute infection, as the oxygen saturation in 72.2% of patients did not decline below 94%, and regarding the inflammatory biomarkers, they were found to be mildly elevated in all cases, despite these elevations not being too high compared with the average normal reference (Table 8). Tawakul et al. reported that serum abnormalities were noticed in 41.90% of the patients [24].

It is clear from our results that the nerve conduction velocities are lower among the severe COVID-19 group and those with inflammatory storms in the majority of the studied nerves. Additionally, the amplitudes are lower among this group for the lower limbs’ nerves mainly (Table 9). This may reflect confounding factors, which are either the superadded critical illness neuropathy or the compression that occurred as a result of a long hospital stay and bed-ridden state, in addition to the co-existing diabetes mellitus and other medical morbidities, which might be the predisposing factors for increased disease severity [10]. In consideration of the correlation between the nerve conduction velocities among cases in COVID-19 groups and specific inflammatory biomarkers, the results showed an inverse relationship between median, peroneal, and tibial motor nerves bilaterally with all studied biomarkers, with higher strength for peroneal nerves, especially with the level of C-reactive protein and LDH. But for both ulnar motor and sensory nerves, the negative correlation is only noticed between the velocity and the N/R ratio. On the other hand, the sural nerve showed a solid inverse relationship with the N/L ratio and a weaker connection with the other biomarkers (Table 10). There were no other studies found by the authors during their survey at the time of writing this research that linked the level of inflammatory biomarkers and the nerve conduction velocities in order to compare these findings.

The main limitations of this study are the retrospective nature of the design as well as the small sample size of the non-COVID-19 neuropathy cases, in addition to the lack of many details at the time of diagnosis of COVID-19 infection and the dependence on medical record findings. Furthermore, we cannot assess the electrodiagnostic findings among all patients in the two groups, and we depend only on the positive GBS and CIDP diagnoses.

## Conclusions

To sum up, COVID-19 significantly increases the incidence of inflammatory polyneuropathy, especially the Guillain-Barre syndrome, and the AIDP with classic motor sensory involvement is the most common type. Although the occurrence of inflammatory polyneuropathy is more common among the mild-to-moderate severity groups, the nerve conduction study parameters are more affected among the severe-to-critical group and those with inflammatory storms.

We recommend active surveying and maybe screening programs for those who recovered from COVID-19 and developed neurological symptoms, as well as increasing doctors' and patients’ awareness about these disorders and not referring to the fatigue and walking difficulties as trivial post-COVID-19 manifestations.
